# Therapeutic benefit of lentiviral-mediated neonatal intracerebral gene therapy in a mouse model of globoid cell leukodystrophy

**DOI:** 10.1093/hmg/ddu034

**Published:** 2014-01-23

**Authors:** Annalisa Lattanzi, Camilla Salvagno, Claudio Maderna, Fabrizio Benedicenti, Francesco Morena, Willem Kulik, Luigi Naldini, Eugenio Montini, Sabata Martino, Angela Gritti

**Affiliations:** 1 Division of Regenerative Medicine, Stem Cells and Gene Therapy, San Raffaele Scientific Institute, San Raffaele Telethon Institute for Gene Therapy (TIGET), Via Olgettina 58, Milano20132Italy; 2 Department of Chemistry, Biology and Biotechnologies, University of Perugia, via del Giochetto, Perugia, Italy; 3 Laboratory for Genetic Metabolic Diseases, Academic Medical Center, University of Amsterdam, F0-224, PO Box 22700, Amsterdam 1100 DE, The Netherlands; 4 Present address: Genethon, 1-bis Rue de l'Internationale, Evry, France

## Abstract

Globoid cell leukodystrophy (GLD) is an inherited lysosomal storage disease caused by β-galactocerebrosidase (GALC) deficiency. Gene therapy (GT) should provide rapid, extensive and lifetime GALC supply in central nervous system (CNS) tissues to prevent or halt irreversible neurologic progression. Here we used a lentiviral vector (LV) to transfer a functional GALC gene in the brain of Twitcher mice, a severe GLD model. A single injection of LV.GALC in the external capsule of Twitcher neonates resulted in robust transduction of neural cells with minimal and transient activation of inflammatory and immune response. Importantly, we documented a proficient transduction of proliferating and post-mitotic oligodendroglia, a relevant target cell type in GLD. GALC activity (30–50% of physiological levels) was restored in the whole CNS of treated mice as early as 8 days post-injection. The early and stable enzymatic supply ensured partial clearance of storage and reduction of psychosine levels, translating in amelioration of histopathology and enhanced lifespan. At 6 months post-injection in non-affected mice, LV genome persisted exclusively in the injected region, where transduced cells overexpressed GALC. Integration site analysis in transduced brain tissues showed no aberrant clonal expansion and preferential targeting of neural-specific genes. This study establishes neonatal LV-mediated intracerebral GT as a rapid, effective and safe therapeutic intervention to correct CNS pathology in GLD and provides a strong rationale for its application in this and similar leukodystrophies, alone or in combination with therapies targeting the somatic pathology, with the final aim of providing an effective and timely treatment of these global disorders.

## INTRODUCTION

Globoid cell leukodystrophy (GLD), or Krabbe disease, is an autosomal recessive lysosomal storage disease (LSD) caused by mutations in the galactocerebrosidase (GALC) gene leading to deficiency of the enzyme β-galactocerebrosidase, a key enzyme in the catabolism of myelin-enriched sphingolipids. The consequent buildup of undegraded substrates results in widespread demyelination and neurodegeneration of the central and peripheral nervous system (CNS and PNS) (1,2). In particular, the lysolipid galactosylsphingosine (psychosine) accumulates at high levels in the CNS of GLD patients when compared with healthy individuals (3) and is considered a major player in the pathogenic cascade (4). Clinically, the disease manifests early in infancy and results in a severe neurological dysfunction that often leads to death by 2 years of age (5). At present, the only clinical treatment for GLD is hematopoietic cell transplantation (HCT). It is beneficial if performed before the onset of symptoms, but its efficacy in correcting the severe neurological disease is variable (6,7). One of the possible reasons underlying the unsatisfactory CNS treatment following conventional HCT, particularly in the rapidly progressive infantile forms, is that the time required to obtain extensive CNS microglia reconstitution from donor-derived myeloid progenitors hampers the possibility to provide therapeutically relevant levels of enzyme in the time window of postnatal CNS development during which disease progression is faster. Indeed, studies performed in animal models (8,9) and in GLD-affected children (10) have documented a disease-driven enhancement of neuronal and oligodendroglial toxicity in the early postnatal CNS. Thus, early therapeutic intervention is crucial to prevent or halt the irreversible neurologic progression and should provide a life-long supply of therapeutically relevant enzyme levels.

Gene therapy (GT) approaches based on intracerebral injection of viral vectors coding for the missing enzymes aim to stably transduce neural cells that would thus become a permanent source of functional proteins (11). Importantly, gene transfer can grant supraphysiological levels and increased secretion of lysosomal enzymes from transduced cells, leading to enhanced enzyme availability through diffusion, cerebrospinal fluid (CSF) flow and axonal transport (12,13). Of note, re-uptake of functional lysosomal enzymes by endogenous enzyme-deficient cells (cross-correction) enhances metabolic improvement, thus reducing the need of widespread vector delivery.

Several pre-clinical studies have shown GALC expression and variable clinical–pathological amelioration in the Twitcher (Twi) mouse (a GALC mutant that recapitulates the severe form of GLD) upon hematopoietic (14), neural (15) and mesenchymal (16) stem cell transplant, intracerebral GT using adeno-associated vectors (AAV) (17,18) and lentiviral vectors (LV) (19), or combination of therapies (20–24). Gene therapy studies highlighted that vector distribution and persistence of transgene expression upon intracerebral delivery largely depend upon the vector tropism and dose, the number of injections and the targeted regions. A proper combination of these factors improves therapeutic benefit while reducing unwanted complications. In this view, our group and others have shown that targeting highly interconnected brain regions facilitates vector and transgene dispersion from one or few injection sites, thus lowering vector load and reducing acute toxicity (12,19).

Immune responses decreasing the efficacy of the strategy and risks related to insertional mutagenesis are major hurdles associated with intracerebral GT using AAV and LV, respectively (25,26). Despite the immunoprivileged status of the nervous tissue, vector and/or transgene-driven immune responses have been documented in animal models treated with multiple intracerebral injections of AAV (27,28) and in clinical studies applying the same approach in patients affected by genetic neurodegenerative diseases (29,30). Owing to lack of preexisting immunity, LV do not trigger significant immune response after delivery into the nervous system, thus ensuring stable expression of therapeutic proteins (31,32). Thus far, clinical application of LV to treat infantile forms of leukodystrophies has been restricted to *ex vivo* hematopoietic stem cell (HSC) GT approaches, with documented therapeutic benefit (33,34). Importantly, results from these clinical trials have confirmed pre-clinical data obtained in animal models, indicating low oncogenic potential of new generation LV in HSC, regardless of the sustained gene marking that is necessary to ensure therapeutic levels of transgene expression (35). In contrast to the large body of data available for HSC, only one study reports the LV integration profile in adult rodent brain cells following *in vivo* transduction (36). To our knowledge, no information is available in the context of neonatal/early postnatal brain transduction, a relevant setting in view of clinical use of LV-mediated intracerebral GT for neurodegenerative infantile disorders.

In this study, we evaluated whether a single-injection, LV-mediated neonatal intracerebral gene delivery of GALC could provide rapid and long-term benefit in Twi mice, specifically addressing the issues of vector genome and transgene expression persistence, immunotoxicity and insertional mutagenesis. Upon LV.GALC injection in the white matter tracts of the external capsule (EC), we documented rapid and sustained transduction of neurons, astrocytes and oligodendrocytes as well as stable production and widespread diffusion of GALC in CNS tissues. The procedure led to minimal and transient activation of inflammatory and immune responses. The early enzymatic supply provided by neonatal GT ensured partial clearance of storage and moderate reduction of psychosine levels. This translated in amelioration of histopathology and enhanced lifespan. We also demonstrated the applicability and safety of this approach in metachromatic leukodystrophy (MLD) mice. Indeed, long-term studies in wild-type (WT) (unaffected) and in MLD mice revealed stable and sustained LV-mediated transgene expression and enzymatic activity. Finally, integration site analyses on brain tissues of LV-injected animals revealed undetectable genotoxicity.

Our data provide a strong rationale for the application of LV-mediated neonatal intracerebral GT to treat CNS pathology in GLD and similar leukodystrophies.

## RESULTS

### LV transduce neurons, astrocytes and oligodendrocytes in the neonatal brain

**Figure 1.**
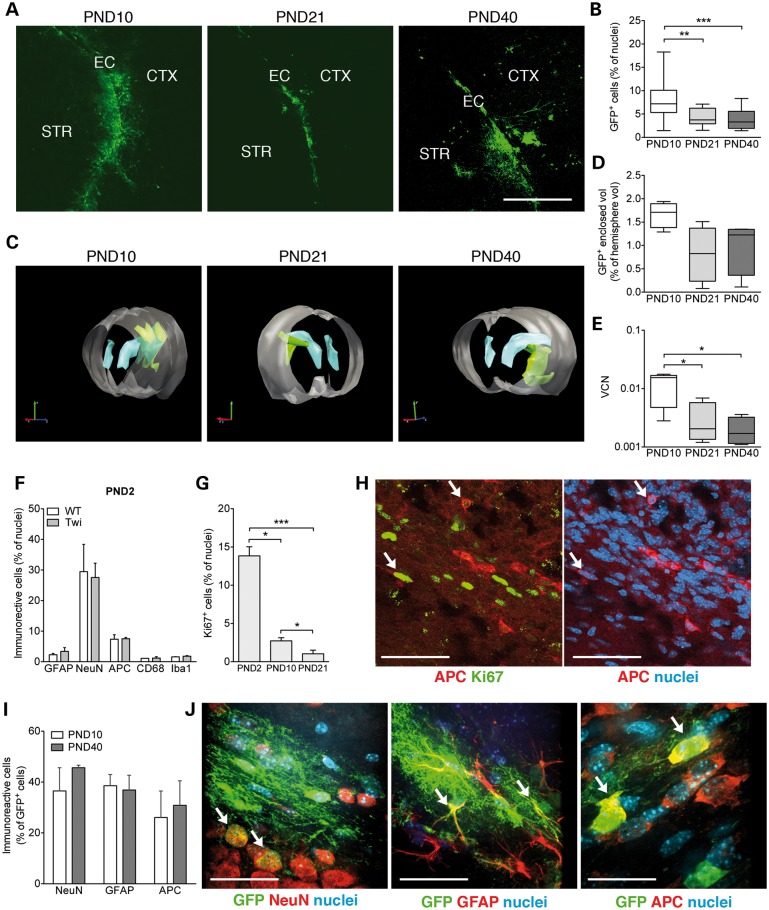
Transgene expression following neonatal bdLV.CTRL injection. Mice were injected at PND2 and analyzed at PND10, PND21 and PND40. Untreated PND2 mice were used as controls. Representative confocal pictures (**A**: EC, external capsule; CTX, cortex; STR, striatum) and quantification (**B**) of GFP^+^ cells. Representative 3D reconstruction (**C**) and quantification (**D**) of GFP^+^ volume (green). Gray, forebrain; light-blue, lateral ventricles. **(E)** Vector copy number (VCN) in the injected hemisphere. Scale bar in A, 300 µm. Data in B, D and E are represented in Box and whiskers plot (min to max), *n* = 4–5 mice/group, 3–5 slices/mouse. (**F**) Cell type composition assessed in EC of PND2 WT (*n* = 4) and Twi mice (*n* = 3). NeuN, neurons; GFAP, astrocytes; APC, oligodendrocytes; CD68 and Iba1, macrophages and microglia. (**G**) Quantification of Ki67^+^ cells in the EC of PND2, PND10 and PND21 mice (*n* = 6 mice/group). (**H**) Representative confocal pictures showing APC^+^ cells (red) expressing the proliferation marker Ki67 (green, arrows) in the EC of PND2 mice. Nuclei counterstained with ToPro (blue). Scale bar, 60 µm. (**I**, **J**) Quantification and representative merged confocal pictures showing the cell type composition of the transduced cell population in the EC of mice analyzed at PND10 and PND40 (*n* = 3 mice/group). Transduced cells are identified by GFP expression; lineage markers in red (NeuN, neurons; GFAP, astrocytes; APC, oligodendrocytes), nuclei counterstained with DAPI (blue). Arrows point to double-labeled cells. Scale bar, 30 µm. In all graphs, data are expressed as mean ± SEM; Kruskall–Wallis test and Dunn's multiple comparison test. *P < 0.05, ** *P* < 0.01, ***P < 0.001.

In order to evaluate the efficiency of transduction and the post-injection kinetics of transgene expression, we injected a bidirectional LV expressing GFP and ΔNGFR (*control vector*, bdLV.CTRL; 2 × 10^6^ TU/1.5 µl) (19) unilaterally in the EC of postnatal day 2 (PND2) WT and Twi mice. By indirect immunofluorescence (IF) analysis using an anti-GFP antibody, we quantified (i) the percentage of GFP^+^ cells (based on GFP expression in cells with defined nuclei) on serial coronal brain sections comprising the injection site and (ii) the GFP^+^ enclosed volume in the injected hemisphere at different time post-injection (PND10, PND21 and PND40). Results indicated considerable numbers of GFP^+^ cells (up to 20% of total cells counted, based on nuclear staining, preferentially located in the EC, striatum and cortex; Fig. 1A and B) and sizeable GFP^+^ enclosed volume (up to 2% of the total hemisphere volume; Fig. 1C and D) in the injected hemisphere at PND10, indicating rapid and efficient LV-mediated transduction of neonatal brain cells. We observed a 40–50% decrease of GFP immunoreactivity between PND10 and PND21 (Fig. 1B and D). The parallel decrease of LV genome (detected by qPCR; Fig. 1E) suggested that cell loss rather than silencing of transgene expression was the mechanism underlying the decrease of GFP expression.

We then assessed which cell types are preferentially targeted by LV upon neonatal injection in the EC by characterizing (i) the cell type composition at the injection site of untreated PND2 WT and Twi mice and (ii) the cell type composition within the GFP^+^ cell population of bdLV.CTRL-treated mice at PND10 and PND40. In the PND2 EC region, ∼3% of total cell counted (based on nuclear staining) were GFAP^+^ astrocytes, 30% were NeuN^+^ neurons, 8% were APC^+^ oligodendrocytes and <2% were CD68^+^ and Iba1^+^ macrophages/microglia, with no significant differences related to genotype (Fig. 1F). In line with the presence of proliferative activity in the neonatal murine CNS, we found that ≈15% of total cell counted (based on nuclear staining) in the PND2 EC (Twi and WT) expressed the proliferation marker Ki67. This percentage significantly decreased with age, being ∼3 and 1% at PND10 and PND21, respectively (Fig. 1G). While neurogenesis is restricted to specific niches in the postnatal CNS (37), glial progenitor cells with mitotic competence, which give rise to astroglia and myelin-producing oligodendrocytes, are widely dispersed throughout the brain, pervading both white and gray matter (38). Of note, 12.4 ± 1.5% (mean ± SEM, *n* = 3 WT mice) and 12.2 ± 4.2% (mean ± SEM*, n* = 3 Twi mice) of the APC^+^ oligodendroglial cells counted in the PND2 EC displayed Ki67 immunoreactivity (Fig. 1H), likely representing proliferating white matter oligodendroglial progenitors that could be proficiently transduced by LV. Indeed, analysis of the cell type composition within the GFP^+^ cell population in bdLV.CTRL-injected mice at PND10 and PND40 indicated robust transgene expression not only in neurons and astrocytes but also in oligodendrocytes (∼30% of total GFP^+^ cells counted; Fig. 1I and J), a cell type that was less frequently transduced following injection in the adult EC (19). The low percentage of macrophages/microglia (CD68, Iba1) expressing GFP (<5% of the total GFP^+^ cells counted; not shown) suggested either occasional LV-mediated transduction of these cell types (which are present in low proportions in the injected area of PND2 mice; Fig. 1F) or residual GFP expression following phagocytosis of GFP-positive cell debris.

### Transduced cells undergo physiologic turnover

**Figure 2.**
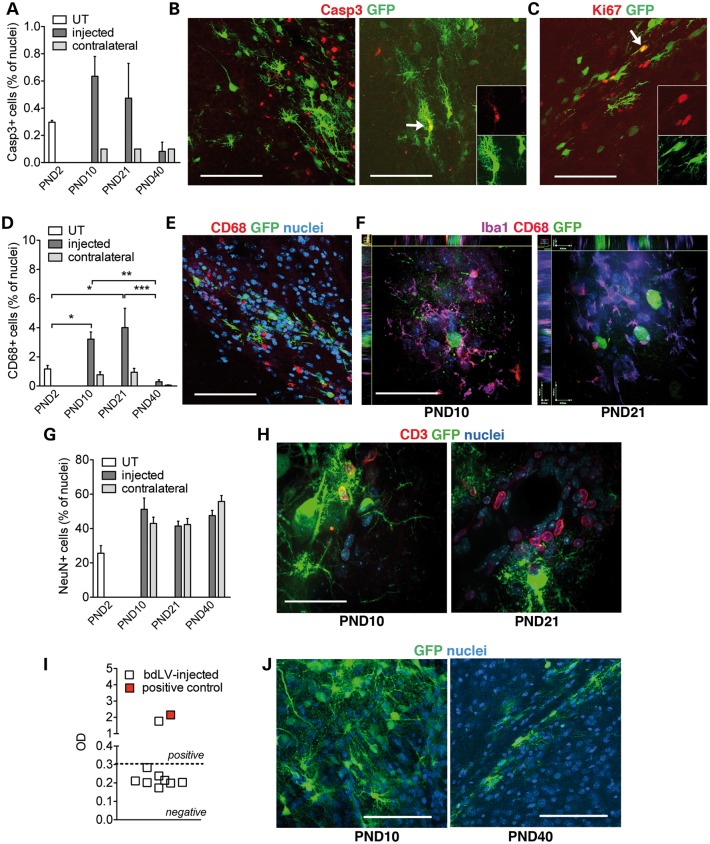
Turnover of transduced cells in the injected area. Mice were injected with bdLV.CTRL at PND2 and analyzed at PND10, PND21 and PND40. (**A**) Apoptotic cells (Casp3^+^) in the injected and in the contralateral (contra), non-injected hemisphere (*n* = 2–5 mice/group). Untreated (UT) PND2 mice were used as controls. (**B**) Representative confocal merged pictures of GFP^+^ (green) and Casp3^+^ (red) cells in the injected site at PND10. Arrow identifies a double-labeled cell; single channels are shown in insets. Scale bar, 100 µm. (**C**) Confocal merged picture (single channels shown in insets) taken at PND10 showing a GFP^+^ cell expressing Ki67 (arrow) in the EC of LV-injected mouse. Scale bar, 100 µm. (**D**) Quantification of CD68^+^ cells in the injected and in the contralateral hemisphere (*n* = 3 mice/group). Legend as in A. (**E**) Representative confocal merged pictures of GFP^+^ (green) and CD68^+^ (red) cells at PND10. Nuclei counterstained with ToPro (blue). Scale bar, 100 µm. (**F**) Representative confocal pictures (*Z*-stack) of the injection site at PND10 and PND21 showing GFP^+^ cells (green) surrounded by CD68^+^ (red) and Iba1^+^ cells (violet) close to GFP^+^-transduced cells (green). Scale bars, 30 µm. (**G**) NeuN^+^ cells in the injected and in the contralateral, non-injected hemisphere (*n* = 2–5 mice/group). Untreated (UT) PND2 mice were used as controls. (**H**) Representative confocal pictures (*Z*-stack) of the injection site at PND10 and PND21 showing CD3^+^ lymphocytes (red) close to GFP^+^-transduced cells (green). Scale bars, 30 µm. (**I**) Anti-GFP antibodies in the sera (1 : 10^3^ dilution) of PND40 mice that were injected at PND2 with bdLV.CTRL (*n* = 9; empty squares). Values are expressed as OD. Threshold (dotted line) is set as the average OD measured in UT mice plus three standard deviations (0.307 OD). The serum collected from an immunized Twi mouse was used as positive control (2.149 OD, 1 : 10^3^ dilution; red square). (**J**) Representative pictures of GFP^+^ cells in the injection site of NOD/SCID mice analyzed at PND10 and PND40 (*n* = 3 mice/group, 5 slices/mouse). Scale bar, 80 µm. Data are expressed as mean ± SEM (A, D, G), scatter plot (I), Box and whiskers (min to max)(J); One-way analysis of variance followed by Bonferroni's multiple comparison tests, *P < 0.05, **P < 0.01, ***P < 0.001.

Cell proliferation and apoptosis contribute to physiological tissue remodeling during early postnatal CNS development and might contribute to the turnover of transduced cells following neonatal LV injection. In the EC of PND2 mice, we found ∼0.3% of total cells counted (based on nuclear staining) expressing cleaved-caspase 3 (Casp3), a marker of apoptosis (Fig. 2A). This percentage moderately and transiently increased at PND10 and PND21 in bdLV.CTRL-injected mice with respect to contralateral EC (Fig. 2A and B). Interestingly, 4.6 ± 2.1% of the total Casp3^+^ cells and 4.5 ± 1.2% of the Ki67^+^ cells in the injected EC expressed GFP at PND10 (mean ± SEM; *n* = 5 mice, 3 slices/mouse; a total of 227 Casp3^+^, 514 Ki67^+^ cells and 1323 GFP^+^ cells were counted; Fig. 2B and C). These results strongly suggested that LV-transduced cells undergo proliferation and apoptosis associated with physiological postnatal brain development. We never observed hyperproliferation of transduced cells (based on histological analysis and Ki67 expression) or formation of tumor masses in mice analyzed at PND40 and at 6 months post-injection.

The transient increase (peaking between PND10 and PND21) of apoptotic cells and of macrophages/microglia that often surround and enwrap GFP^+^ cells in or close to the injection site (Fig. 2D–F) was likely an acute reaction to the injection procedure (it was not observed in the contralateral hemisphere), being completely normalized at PND40 (Fig. 2A and D). The minimal toxicity of the procedure was also confirmed by the absence of neuronal death, as indicated by the comparable size of the NeuN^+^ cell population in the injected and in the contralateral hemispheres (Fig. 2G), with negligible percentages of apoptotic NeuN^+^ cells (<1% of the total cells counted; not shown).

A variable (from animal to animal) but overall moderate and transient infiltration of CD3^+^ lymphocytes was observed at PND10 and PND21 close to the injection site in both Twi and WT mice (Fig. 2H), suggesting a potential contribution of the adaptive immunity in the clearance of GFP^+^ cells, possibly related to the occasional leakage of LV particles to the periphery as a result of the injection procedure. However, anti-GFP antibodies were detected only in one of nine bdLV.CTRL-injected mice analyzed at PND21 and PND40 (Fig. 2I). Also, when we injected bdLV.CTRL in neonatal NOD/SCID mice, which have a severe combined immunodeficiency affecting T- and B-lymphocyte development, we still observed a consistent ≈30% decrease in the percentage of GFP^+^ cells within the first 3 weeks of age (6.2 ± 0.9, 4.4 ± 0.3 and 4.2 ± 0.8% of GFP^+^ cells at PND10, PND21 and PND40, respectively; mean ± SEM, *n* = 3–4 mice/group; Fig. 2J). Overall these results suggested minimal immune response against GFP-expressing cells.

### Injection of bdLV.GALC in the neonatal EC allows fast, widespread and stable restoration of enzymatic activity

**Figure 3.**
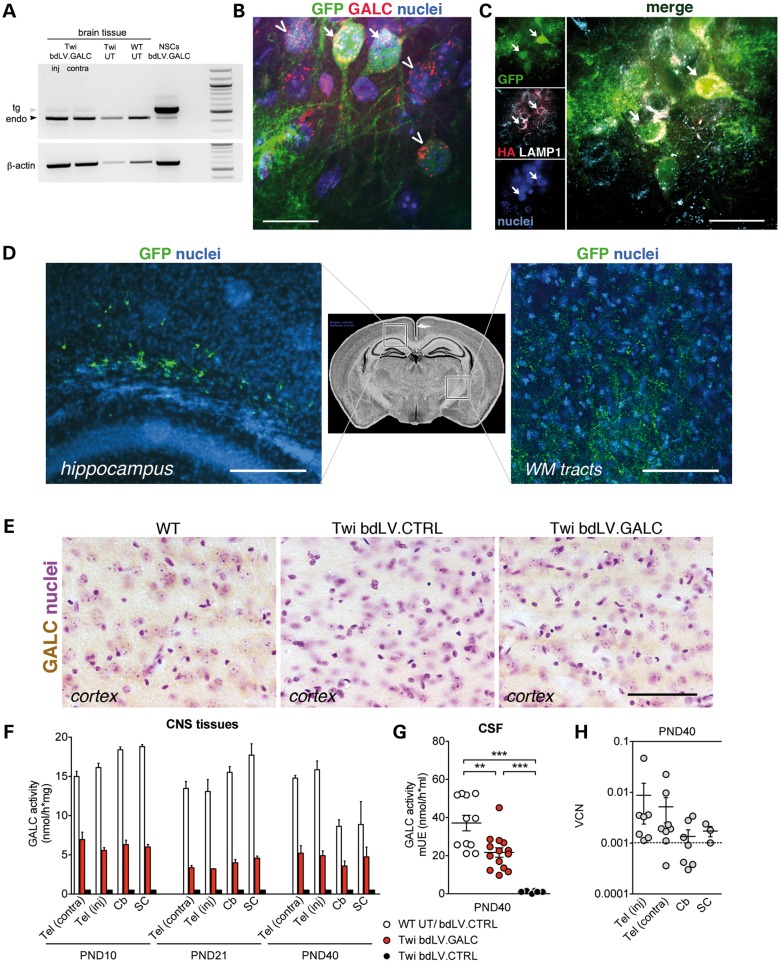
GALC expression and activity in CNS tissues of bdLV.GALC-treated mice. (**A**) RT–PCR using two pairs of primers designed on a sequence of the bdLV cassette (expected size 550 bp; gray arrowhead, tg) and on a sequence of the endogenous GALC (expected size 470 bp; black arrowhead, endo). Tg GALC mRNA is present in the injected (inj) and contralateral (contra) hemisphere of bdLV.GALC-injected Twi mice. β-actin was used as normalizer. UT, untreated mice; bdLV.GALC-transduced neural stem cells (NSCs) (15) were used as positive control. (**B**) Representative confocal merged picture showing GALC^+^GFP^+^-transduced cells (arrows) and GALC^+^GFP^−^ cross-corrected cells (arrowheads). Nuclei counterstained with DAPI (blue). Scale bar, 15 µm. (**C**) Representative confocal pictures (single channels and merged pictures) showing co-localization of HA (red) and LAMP-1 (white) signal in GFP^+^ cells (arrows). Nuclei counterstained with DAPI (blue). Scale bar, 15 µm. (**D**) Representative confocal pictures showing GFP^+^cells and fibers caudally to the injection site, at the level of the hippocampus and in the posterior white matter (WM) tracts (boxed regions in the low magnification schematic coronal section) in PD40 mice following neonatal injection of bdLV.GALC. Scale bar, 150 µm. (**E**) Immunohistochemistry using an anti-GALC antibody shows the diffused presence of the GALC protein (brown) in cortical regions of WT and bdLV.GALC-treated Twi mice (PND40). Tissues of bdLV.CTRL-injected Twi littermates were used as negative control. Scale bar, 100 µm. (**F**) GALC activity measured at PND10, PND21 and PND40 in CNS tissues of WT and Twi mice injected with bdLV.CTRL and bdLV.GALC. *n* = 3–10 mice/group. (**G**) GALC activity in the cerebrospinal fluid (CSF) of UT or bdLV.CTRL-injected WT mice, bdLV.CTRL- and bdLV.GALC-injected Twi mice (*n* = 6–14 mice/group). Data in F and G are expressed as mean ± SEM. One-way analysis of variance and Bonferroni's multiple comparison tests. In F, for each age, bdLV.GALC samples are significantly different (*P* < 0.05) from both WT UT/bdLV.CTRL and Twi bdLV.CTRL samples (not indicated). In G, **P < 0.01 and ***P < 0.001. (**H**) VCN in CNS tissues of mice injected with bdLV.CTRL or bdLV.GALC (*n* = 4–8 mice/group). Values of ≤10^−3^ (dotted line; corresponding to Ct < 36) are considered undetectable. Data are expressed as mean ± SEM. One-way analysis of variance and Bonferroni's multiple comparison tests (*P* = 0.5644). Tel, telencephalon; Cb, cerebellum; SC, spinal cord. Inj, injected hemisphere; contra, contralateral non-injected hemisphere.

We next injected a bdLV expressing GFP and GALC-HA (*therapeutic vector*, bdLV.GALC; 2 × 10^6^ TU/1.5 μl) (19) unilaterally in the EC of PND2 Twi mice and assessed GALC expression and activity in CNS tissues at PND10, PND21 and PND40. Untreated (UT) or bdLV.CTRL-injected littermates served as controls. By RT–PCR (Fig. 3A) and confocal IF analysis (Fig. 3B and C), we detected the expression of GALC-HA at the injection site of bdLV.GALC-treated Twi mice. GALC or HA expression was undetectable by IF in bdLV.CTRL-treated Twi mice and barely detectable in UT and bdLV.CTRL-treated WT mice (not shown), further confirming overexpression of this lysosomal enzyme in transduced (GFP^+^) cells (Fig. 3B). The transgenic GALC-HA protein was present in lysosomes, as demonstrated by co-localization with LAMP-1 (Fig. 3C). Importantly, we found a sizeable population of putative cross-corrected cells (GALC^+^GFP^−^) close to GALC-overexpressing transduced cells (Fig. 3B).

A small number of GFP^+^-transduced cells and abundant GFP^+^ fibers were detected rostrally and caudally to the injection site (ipsilaterally), typically in the hippocampal region and in posterior white matter tracts (Fig. 3D). GFP^+^ fibers were also detected in the contralateral hemisphere (not shown), confirming axonal GFP transport (19). In line with this result, diffused GALC expression was shown by IHC in CNS regions of bdLV.GALC-injected mice that are devoid of transduced cells (Fig. 3E; cortex), suggesting widespread enzyme transport. Accordingly, GALC-specific activity was measured not only in the telencephalon (Tel; injected and contralateral hemisphere) but also in the cerebellum (Cb) and spinal cord (SC) of bdLV.GALC-treated Twi mice (≈40% of physiological levels), as early as PND10, with no significant time-dependent differences (Fig. 3F). Importantly, enzyme activity at 50% of physiological levels was detected in the CSF of bdLV.GALC-treated Twi mice (Fig. 3G; PND40), indicating stable enzyme secretion from transduced cells. We did not find anti-HA antibodies in the serum of bdLV.GALC-injected mice analyzed at PND40 (not shown). Whereas LV genome was consistently present at higher levels in the injected region, detectable vector copy number (VCN) was found in the contralateral hemisphere and occasionally in the Cb and SC (Fig. 3H), suggesting sporadic spreading of LV particles upon neonatal injection, possibly through diffusion and/or transport along white matter tracts.

### Reduction of toxic storage, amelioration of pathology and prolonged lifespan in bdLV.GALC-treated Twi mice

**Figure 4.**
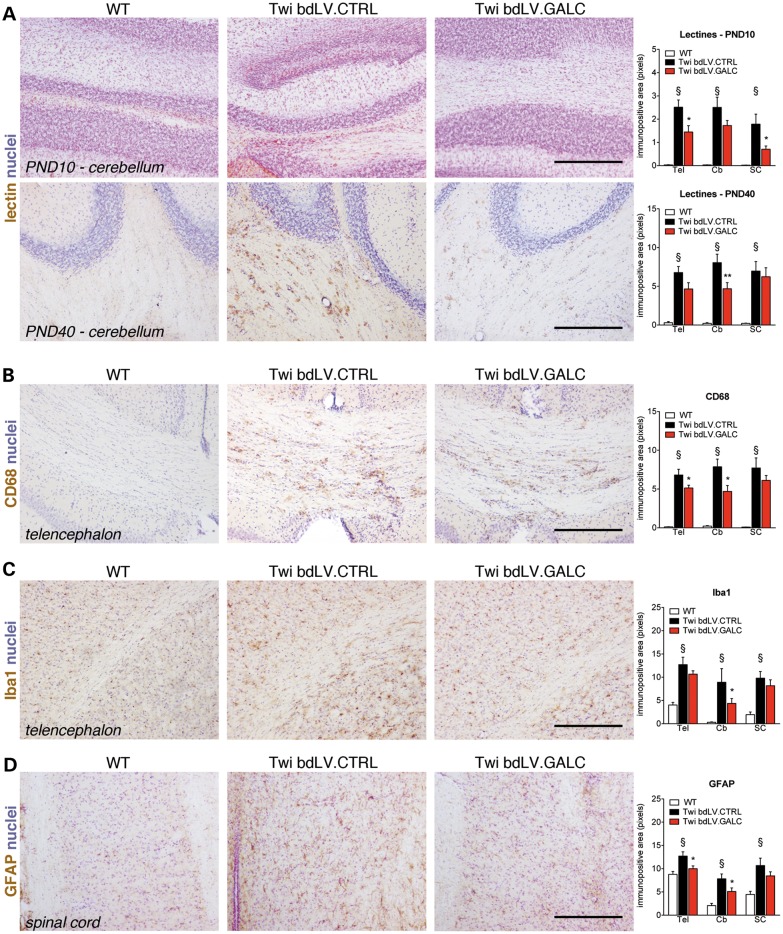
Neonatal bdLV.GALC injection reduces galactolipid storage and ameliorates pathology. (**A**–**D**) Representative pictures from different CNS regions (forebrain, cerebellum and spinal cord) and quantitative analysis of lectins (A; Cb, PND10 and PND40), CD68 (B; forebrain, PND40), Iba-1 (C; forebrain, PND40) and GFAP (D; spinal cord, PND40) from UT WT mice and from bdLV.CTRL- and bdLV.GALC-injected Twi littermates. Scale bars, 500 µm. Data are expressed as mean ± SEM. *n* = 3–4 mice/group, 3 slices/brain region. Two-way analysis of variance followed by Bonferroni's multiple comparison tests. § *P* < 0.05 versus WT; *P < 0.05, **P < 0.01 versus Twi bdLV.CTRL.

**Figure 5.**
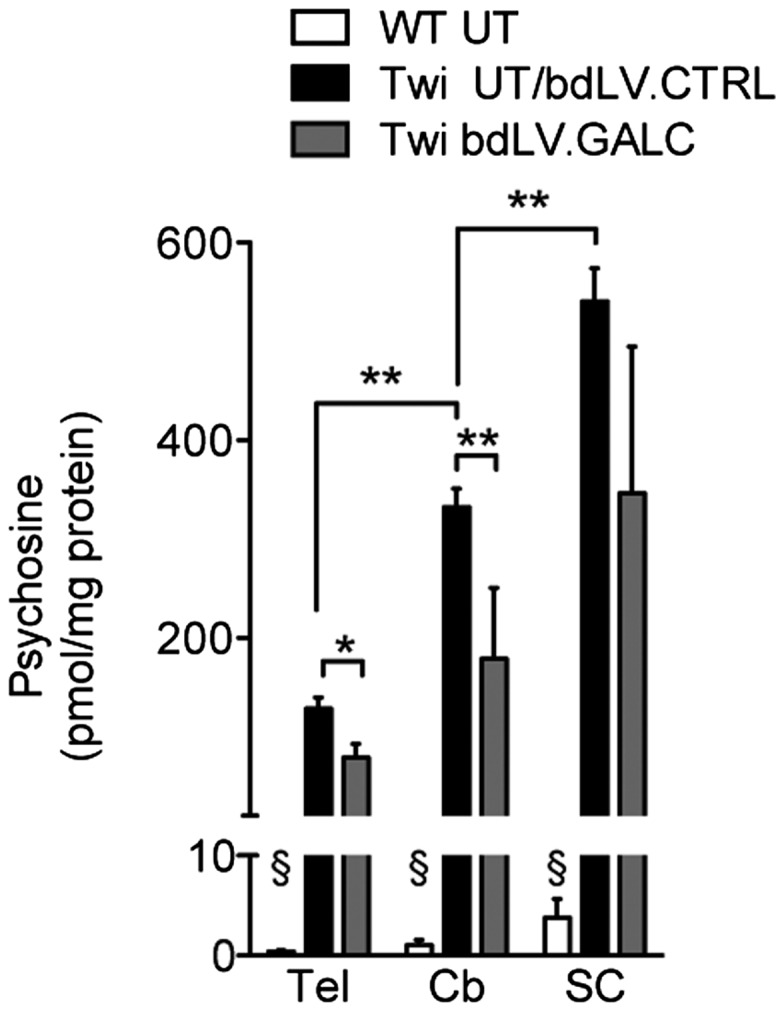
Neonatal bdLV.GALC injection reduces psychosine accumulation. Psychosine levels are significantly increased in the Tel, Cb and SC of UT Twi mice as compared with WT littermates at PND40. Neonatal injection of bdLV.GALC in Twi mice reduces psychosine accumulation by 25–40% in the CNS regions analyzed at PND40. *n* = 3–10 mice/group; data expressed as mean ± SEM. One-way ANOVA with Dunnet multiple comparison test; § *P* < 0.001 versus UT Twi; *P < 0.05, **P < 0.01.

In order to evaluate whether the enzymatic supply provided to CNS tissues by LV-mediated neonatal GT reduced storage and ameliorated histopathology, we performed lectin histochemistry (39) and IHC using antibodies against GFAP (astroglia), Iba-1 and CD68 (microglia and macrophages) on matched brain sections derived from bdLV.CTRL- and bdLV.GALC-treated Twi mice as well as from UT WT littermates. Quantification of lectin-immunopositive regions in the Tel, Cb and SC indicated a significant increase of galactolipid storage in bdLV.CTRL-treated Twi mice when compared with WT littermates (Fig. 4A). In agreement with our previous studies (9), lectin^+^ cells were mainly localized in the white matter and were present early postnatally (Fig. 4A; PND10), progressively increasing with age (Fig. 4A; PND40). Of note, storage was significantly decreased in some of the CNS regions examined in bdLV.GALC-treated Twi mice at PND10 and PND40, suggesting rapid and persistent clearance operated by the transgenic GALC enzyme. The moderate but consistent reduction of CD68 and Iba1 immunoreactivity (Fig. 4B and C) in the Tel and Cb of bdLV.GALC-treated Twi mice with respect to bdLV.CTRL-treated littermates further indicated clearance of tissue storage and/or reduced recruitment/activation of macrophage-derived globoid cells and microglia. The availability of functional enzyme was likely responsible for the reduction of astrogliosis in CNS tissues of bdLV.GALC-treated Twi mice (Fig. 4D), either through clearance of storage in astrocytes or indirectly, through the reduction of the pro-inflammatory environment sustained by activated macrophages and microglia. Psychosine accumulates in the brain of Twi mice starting from the early postnatal days, and its levels progressively increase over time, peaking at the late stages of disease progression (9). We found a rostral to caudal increase in the levels of psychosine in the CNS of UT Twi mice at PND40 (up to 200- to 300-fold the physiological levels) (Fig. 5). These levels were reduced by 25–40% in bdLV.GALC-treated Twi littermates (Fig. 5), indicating the activity of the transgenic GALC in the removal of this toxic lysolipid metabolite. The minor amelioration observed in the SC of treated mice when considering any of the parameters studied is likely reflecting the higher degree of tissue damage that characterizes this region when compared with the Cb and Tel, in agreement with the caudal-to-rostral onset and progression of the disease in this GLD mouse model (40).

**Figure 6.**
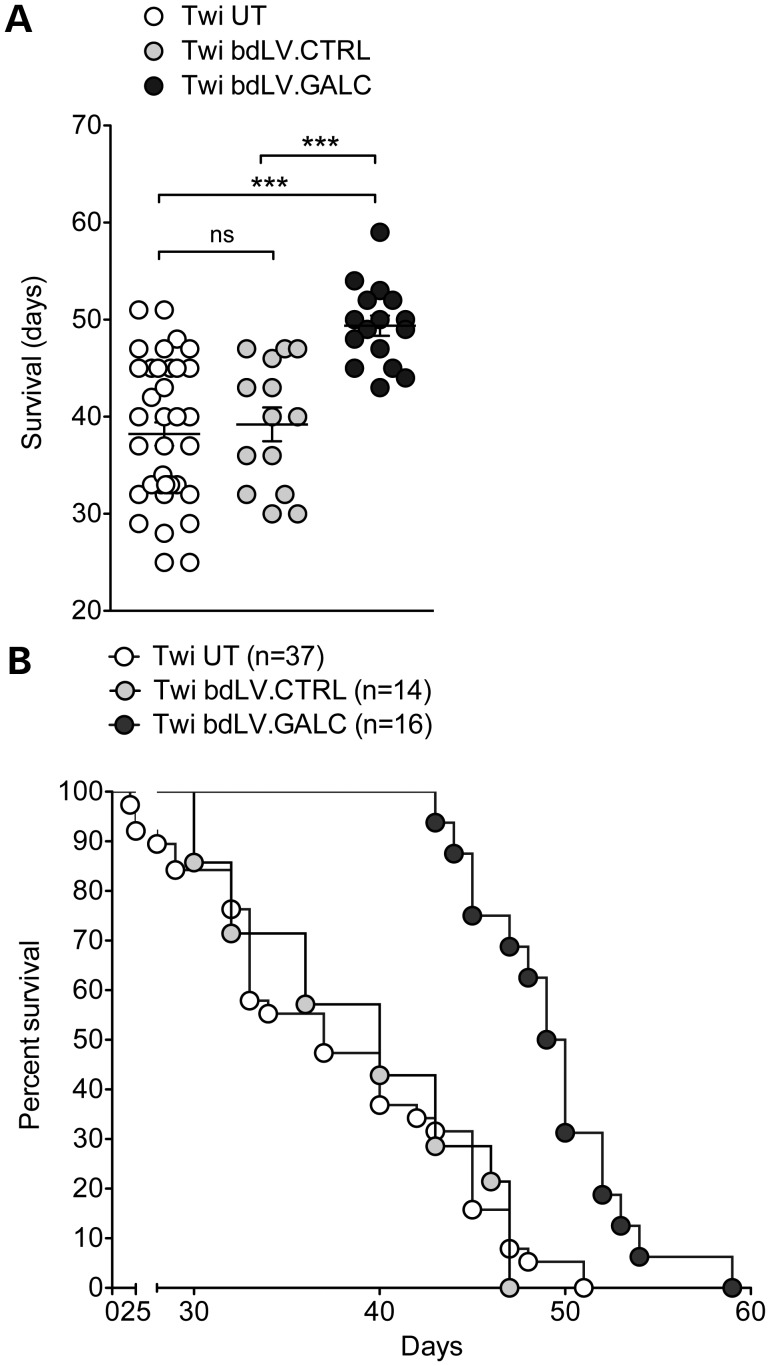
Neonatal gene therapy delays the onset of symptoms and prolongs survival of Twi mice. Average survival (**A**; mean ± SEM) and Kaplan-Meier survival curve (**B**) of bdLV.GALC-treated (*n* = 16), untreated (UT) (*n* = 37) and bdLV.CTRL-treated (*n* = 14) Twi mice. One-way ANOVA with Dunnet's multiple comparison test, ***P < 0.001 (A) Log-rank (Mantel–Cox) test (B), *P* = 0.93 (Twi UT versus Twi bdLV.CTRL) and *P* < 0.0001 (Twi UT versus Twi bdLV.GALC and Twi bdLV.CTRL versus Twi bdLV.GALC).

Of note, the modest amelioration of histopathology and the partial reduction of psychosine accumulation consequent to enzymatic correction resulted in functional benefit. Indeed, bdLV.GALC-treated Twi mice showed delayed onset of symptoms and moderate but significant increase of lifespan (median survival, 49.5 days) when compared with both UT (median survival, 39 days) and bdLV.CTRL-treated littermates (median survival, 41 days; Fig. 6A and B).

### Long-term transgene expression in CNS tissues of LV-injected mice

We next assessed whether LV-mediated neonatal GT may provide stable transduction and long-term transgene expression coupled to a safe profile of LV distribution. As the survival of bdLV.GALC-injected Twi mice (maximum 60 days; see Fig. 6) hampers to assess long-term expression of the enzyme, for these experiments we took advantage of WT littermates and of MLD mice, the latter having a normal lifespan despite undetectable activity of the lysosomal enzyme Arylsulphatase A (ARSA) (19). Our previous work demonstrated comparable protein distribution and metabolic correction after EC injection of bdLV.GALC and bdLV.ARSA in Twi and MLD mice, respectively (19), providing a rationale for performing long-term GT studies in this relevant disease model.

**Figure 7.**
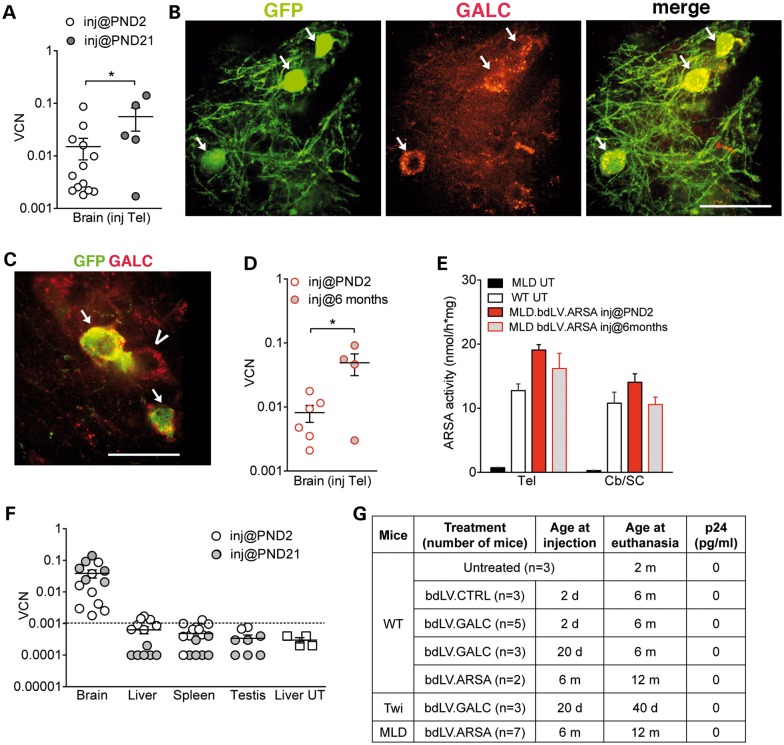
Persistence of viral genome and transgene expression in CNS tissues of bdLV-injected mice. (**A**) VCN in the injected hemisphere of 6-month-old WT mice injected with bdLV.CTRL or bdLV.GALC at PND2 and PND21. Student's *t*-test, *P < 0.05. (**B**, **C**) Representative confocal picture showing transduced cells (GFP^+^GALC^+^, arrows) and cross-corrected cells (GFP^−^GALC^+^, arrowheads) at the injection site 6 months post-injection. Green, GFP; red GALC. Scale bars, 30 µm (B) and 20 µm (C). (**D**) Detectable VCN (>10^−3^) measured 6 months post-injection in the telencephalon (Tel) of MLD mice injected with bdLV.ARSA at PND2 or at 6 months. Data are expressed as mean ± SEM. Student's *t*-test, *P < 0.05. (**E**) ARSA activity measured 6 months post-injection in CNS tissues (Tel, telencephalon; Cb/SC, cerebellum/spinal cord) of MLD mice, either untreated (UT) or injected with bdLV.ARSA at PND2 and at 6 months of age. 12-month-old UT WT littermates served as controls. Data are expressed as mean ± SEM. *n* = 3–9 mice/group. Two-way analysis of variance followed by Bonferroni's multiple comparison tests posttest. WT versus LV-injected MLD, *P* > 0.05. (**F**) VCN measured in the brain and peripheral organs of WT mice injected at PND2 and PND21 with bdLV.CTRL or bdLV.GALC. The liver of UT mice was used as negative control. Values of <10^−3^ (dotted line; corresponding to Ct > 36) are considered undetectable. (**G**) The p24 LV capsid protein is undetectable in the serum of bdLV-injected mice, regardless of the age at injection, the age at euthanasia and the vector used.

We first injected bdLV.CTRL and bdLV.GALC in the EC of PND2 and PND21 WT mice and analyzed the animals at 6 months of age, in order to compare the long-term outcome of neonatal versus adult injection. Integrated LV genome was measured in the injected hemisphere of all bdLV-injected mice (Fig. 7A). The lower VCN measured in PND2- injected as compared with PND21-injected mice likely reflects the partial clearance of transduced cells owing to postnatal brain tissue remodeling. The numerous GFP^+^GALC^+^ cells found in the EC of bdLV.GALC-injected mice (Fig. 7B) and the presence of GFP^−^GALC^+^ cells (Fig. 7C) indicated that the transgenic enzyme was stably produced at supraphysiological levels by transduced cells, secreted and recaptured by surroundings cells, thus ensuring cross-correction.

We next injected bdLV.ARSA in the EC of PND2 and 6-month-old MLD mice, analyzing animals at 6 months post-injection. We found integrated LV genome in the injected hemisphere (Fig. 7D) and physiological levels of ARSA activity in the whole CNS of bdLV.ARSA-injected mice (Fig. 7E). Altogether our results provide proof-of-concept that a single injection of a therapeutic LV in the neonatal or adult EC ensures fast, stable and widespread enzymatic correction of CNS tissues in enzyme-deficient mice.

In the long-term evaluation of vector and transgene expression/activity, we examined a total of 50 LV-injected mice. In none of them we found evidence of brain tumor masses (by gross morphological evaluation at necropsy; *n* = 44) or abnormal cell proliferation (*n* = 6 brains examined by histology and by IHC for Ki67 expression).

Finally, we checked for the presence of LV genome in liver, spleen and gonads of WT and MLD mice injected at PND2 or at PND21 and analyzed at 6 months of age. VCN values in these organs were in the background range measured in tissues from UT mice (Fig. 7F), with a possible trend for higher values in the PND2-injected group. These results suggested none to occasional leakage of LV particles outside CNS tissue through the circulation following neonatal injection, possibly as a consequence of the elevation of CSF pressure owing to the injection procedure and/or to unpredictable targeting of blood vessels. The p24 antigen (the viral capsid protein) was undetectable in sera of bdLV-treated mice, regardless of the mouse genotype, the age at injection, the vector and the time post-injection (Fig. 7G), thus excluding the presence of LV particles in the circulation.

### Profile of LV integration in CNS tissues

**Figure 8.**
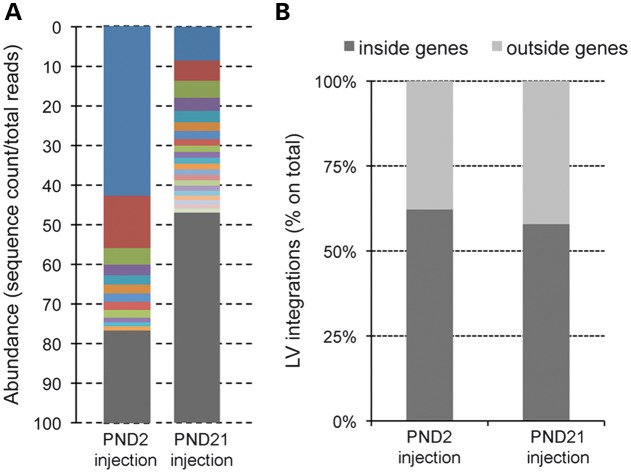
LV integration profile in CNS tissues following intracerebral injection in neonates and adult mice. (**A**) Clonal abundance calculated as the proportion of sequencing reads representing each integration site within each data sets. Gray bars represent all vector integrations with an abundance of <1%. (**B**) Relative proportions of LV integrations mapping inside and outside genes in the two data sets (PND2 and PND21).

In order to assess a potential genotoxic effect of LV-mediated GT, we characterized the genomic integration profile of the LV constructs used in this study in brain tissues of mice injected at PND2 or at PND21 and analyzed at PND40 (Table 1). We amplified the vector genomic junctions by linear amplification-mediated (LAM)-PCR (41). The LAM-PCR products sequenced by 454-pyrosequencing were mapped on the mouse genome (mm9) using a dedicated bioinformatics pipeline. Overall, we univocally mapped 4,161 (PND2 injection) and 16,549 (PND21 injection) integration sites. After filtering for redundancy, the remaining unique integration sites were 178 (4%) and 962 (6%) from mice injected at PND2 and PND21, respectively (Table 1). The distribution of the LAM-PCR bands (Supplementary Material, Fig. S1) as well as the proportion of sequencing reads representing each integration site within each data sets (a surrogate readout for the relative abundance of vector-marked cell clones in brain tissues of LV-injected mice; Fig. 8A and Supplementary Material, Table S1) showed a pattern of polyclonal marking in the PND21-injected mice and a trend toward oligoclonality in the PND2-injected mice. Common insertion sites (CIS) are integration hotspots that may represent an early readout of insertional mutagenesis or an intrinsic bias of the vector of interest. By means of a specific analysis (42), we identified a single CIS in a 56-Kb region encompassing the *galc* gene that was targeted by 17 integrations, exclusively in exons and only in bdLV.GALC-injected mice. In agreement with a previous report on LV-injected adult brain tissues (36), we found that ∼60% of LV integrations mapped within genes (Fig. 8B). Also, Gene Ontology analysis showed preferential targeting of genes related to neural/neuronal function, irrespectively of the vector used or of the age at injection (Supplementary Material, Table S2).

**Table 1.** Genomic integration profile of LV constructs in brain tissues

Vector	Number of mice (VCN in injected tissues)	Genotype	Number of total reads	Number of unique genomic integration sites	Age at injection
bdLV.CTRL	3 (0.35; 0.22; 0.53)	Twi	3,415	203	PND21
bdLV.GALC	3 (0.12; 0.27; 0.43)	Twi	11,137	676	
bdLV.ARSA	2 (0.09; 0.046)	MLD	1,027	7	
	1 (0.055)	WT	970	76	
			*16,549*	*962*	
bdLV.CTRL	2 (0.0021; 0.0012)	Twi	72	20	PND2
bdLV.GALC	1 (0.0011)	Twi	4,089	158	
			*4,161*	*178*	

The genomic integration profile of the different LV constructs used in this study (bdLV.CTRL, bdLV.GALC and bdLV.ARSA) was analyzed in brain tissues of PND40 mice (WT, Twi and MLD) that were injected at PND2 and at PND21. Vector copy number measured in the injected tissue of each animal is shown in brackets. Overall, we univocally mapped 4,161 and 16,549 integration sites from brain tissues of mice injected at PND2 and PND21, respectively (total reads). After filtering for redundancy, the remaining unique genomic integration sites were 178 and 962, respectively.

## DISCUSSION

This study shows the efficacy of a single intracerebral injection of LV.GALC in a highly interconnected white matter region of GALC-deficient neonates in achieving fast, widespread and stable reconstitution of enzymatic activity. This translates into partial but relevant removal of toxic storage that leads to amelioration of pathology and enhanced survival. Also, our results upon short- and long-term observation of LV-injected Twi and MLD mice, respectively, strongly suggest that the LV-mediated neonatal GT tested here is not only effective in terms of robust transgene persistence but it is also well tolerated, leading to minimal and transient activation of inflammatory and immune responses and with low genotoxic risk, major issues that have to be considered in the risk assessment of GT strategies.

One of the novel and interesting findings described here is the high percentage of transduced oligodendrocytes (30–40% of the GFP^+^-transduced cells) upon LV injection in the neonatal EC. We have previously shown that oligodendrocytes are less efficiently transduced than neurons and astrocytes upon LV injection in the adult EC (19). The 3- to 4-fold increase in transduced oligodendrocytes reported here is likely related to proficient transduction of proliferating oligodendrocyte progenitors that are present in this region at the time of injection. The ability to transduce proliferating and post-mitotic oligodendroglia distinguishes LV from AAV (43–45) and adds a rationale for the application of a LV-mediated intracerebral GT platform to treat CNS pathology in GLD and similar leukodystrophies, i.e. MLD. In fact, while (supra)physiological levels of the functional enzyme secreted by *in vivo* transduced neurons and astrocytes (or by transplanted cells) can cross-correct enzyme-deficient oligodendrocytes in the mouse brain (9,19,27), this mechanism appears less efficient in large brains (46).

The rapid and robust transgene expression achieved upon neonatal LV injection was followed by a decrease in the total number of transduced cells within the first 3 weeks post-injection. Our data strongly suggest that transduced cells participate to the physiological remodeling that is active in the first postnatal weeks of rodent CNS development. In particular, a fraction of them undergo proliferation and apoptosis, the latter representing a key process occurring in several regions of the neonatal/early postnatal murine (47) and human brain (48) to shape neuronal wiring and synaptic connections. This likely accounts for the transient recruitment/activation of the innate immune cells in the injection site consequent to LV injection, as indicated by the wave of macrophages, some of them in proximity to GFP^+^ cells, suggesting phagocytosis. We cannot exclude that GFP/GALC overexpression (owing to the higher vector: cell ratio consequent to injection in the small neonatal brain) might enhance apoptosis and, in turn, local macrophage-mediated phagocytosis of transduced cells.

In our experimental setting, the contribution of the adaptive immune response in the removal of transduced cells was minimal (if any), despite GFP is a non-mammalian immunogenic protein. We envisage that lack of preexisting immunity to LV components, CNS-restricted and short-range diffusion of LV particles after EC injection and the immunoprivileged status of the neonatal CNS might explain the absence of immune response against GFP. Moreover, the presence of stable LV integration and transgene expression (both GALC and ARSA) in transduced cells at the injection site coupled to the persistent enzymatic activity up to 6 months after neonatal or adult injection (assessed in MLD mice) strongly suggests that no immune response is established against LV-transduced enzyme-expressing cells. Of note, while Twi and MLD mice have no residual protein expression, many GLD and MLD patients have mutations resulting in dysfunctional proteins. This would decrease the chances to build an immune response following intracerebral LV-mediated GT, potentially sparing patients the administration of an immunosuppressive regimen in the perspective of moving this platform into clinical translation.

The transient turnover of transduced cells minimally affected GALC activity, which was widespread and rapidly achieved in Twi mice upon neonatal injection of the therapeutic vector. This translated in partial but overall considerable reduction (despite some region-dependent variability) of a number of pathological hallmarks, including glycosphingolipid storage, psychosine accumulation, astrogliosis and microglia activation/recruitment. Owing to the limited demyelination characterizing the Twi model used in this study (15,40), we could not absolutely document an amelioration of this pathological hallmark upon our treatment.

We previously described correction of CNS pathology in Twi mice that underwent the same GT protocol at PND21, corresponding to the early symptomatic stage (19). However, only the neonatal injection of LV.GALC described here translated in delayed onset of symptoms and in moderate but significant improvement of lifespan. Thus, providing relevant (even if less than physiological) levels of GALC enzymatic activity in CNS tissues early before the onset of symptoms is crucial to slow down the pathology progression and to ensure some therapeutic benefit. The extension of lifespan reported here is moderate and in line with that observed in Twi mice that underwent other CNS-directed treatments, i.e. transplantation of gene-corrected neural stem cells (NSCs) (15) or mesenchymal stem cells (16), intratechal administration of recombinant GALC (49), and likely reflects failure of these approaches to provide sufficient levels of functional enzyme to correct the somatic pathology and particularly the PNS, which is likely the ultimate cause of lethality in this mouse model. In fact, treatments able to correct the systemic disease associated with GLD, such as HCT, alone or in combination with systemic delivery of GALC-expressing vectors (14,20–23) as well as novel HSC GT protocols (50), significantly extend the lifespan of relevant GLD models. However, these approaches hardly achieve timely and consistent metabolic correction of CNS tissues. Indeed, LV as well as the majority of AAV (with few exceptions, such as AAV9) (51) poorly cross the blood–brain barrier upon systemic injection. Also, the variable HC engraftment and the slow turnover of HC-derived myeloid progeny in CNS tissues translate in variable and delayed elevation of enzymatic activity. Recent pre-clinical studies in animal models and clinical evidence in MLD infants suggest that these and other limitations suffered by conventional HCT (i.e. the requirement for compatible donor cells, the high morbidity and mortality associated with the procedure) might be partially overcome by using autologous HSC engineered to express supraphysiological levels of enzyme activity coupled to the use of myeloablative regimens able to deplete functionally defined brain microglial cells (34,52). However, data obtained so far indicate that this treatment benefits MLD patients if performed before or very soon after the onset of symptoms. It is not known whether the same approach might benefit already symptomatic patients or patients with a delayed disease onset. More importantly, it is currently neither known nor predictable whether this HSC gene therapy approach will be applicable to GLD infants. Indeed, despite belonging to the same LSD class and having clinical similarities, MLD and GLD present with peculiar features that might impact on the development of effective treatments. For example, toxicity resulting from GALC overexpression in HSC requires a complex gene transfer vector design to tightly regulate transgene expression (50). So, there is a rationale for the development of alternative or complementary ways to target rapidly, specifically and safely the CNS. The rapid supply of sustained enzymatic levels in the whole CNS provided by LV-mediated intracerebral GT might be of relevance for patients with a specific involvement of the CNS and limited/absent peripheral neuropathy. Also, it might benefit patients in which the rapid disease progression may require urgent enzyme coverage to prevent irreversible CNS damage and/or to buy time while waiting for a delayed therapeutic benefit provided by other treatments. In this perspective, combination of LV-mediated intracerebral GT with therapies able to correct the somatic pathology (i.e. HSC transplant or HSC GT) might succeed in providing effective and timely cure for these global diseases.

A recent paper described extended lifespan of Twi mice treated with a combination of intracerebroventricular, intraparenchymal (Cb) and systemic delivery of AAVrh10 expressing GALC (24). Despite the important therapeutic outcome of this approach, the high number of viral particles that had to be injected in the neonatal brain coupled to two shots of systemic delivery to achieve high transduction of PNS and peripheral organs also raise safety concerns related to AAV-mediated gene transfer. Similar considerations might apply to a novel GT approach based on direct CSF administration of AAV9 that has recently been reported as highly effective in treating the central and somatic pathology of mucopolysaccaridosis IIIA mice (53). Indeed, low but definite risk of integration and insertional mutagenesis, risk of germline transmission and risks related to immune responses to either vector or transgene product are aggravated with the vector dose and its multi-organ diffusion (54).

The single-injection, little invasive LV-mediated GT strategy proposed here ensures fast and stable production of functional enzymes (close to or even slightly above the physiological levels for GALC and ARSA, respectively) from a relatively small pool of endogenous transduced enzyme-overexpressing brain cells and absence of vector in organs that have not been targeted by the GT. Still, one issue related to the use of LV for GT approaches is the risk of oncogenesis linked to insertional mutagenesis. Analysis of vector integrations has revealed highly polyclonal and multi-lineage hematopoiesis resulting from LV-transduced gene-corrected HSC, both in pre-clinical models (35) and in patients enrolled in clinical trials (34,55), indicating a safe integration profile in this clinical setting. However, this issue has never been addressed before in brain tissues upon neonatal/early postnatal LV-mediated transduction. The integration site analysis performed here showed a polyclonal pattern of integration in adult-injected mice, whereas in neonatally injected mice, some integrations were represented by many sequence reads. The tendency to oligoclonality of this data sets might be a bias resulting from the low number of total sequence reads retrieved (owing to the small sample size), which hampers the correct quantification of the clonal abundance. Increasing the number of samples could overcome this bias. On the other hand, we speculate that this pattern may also reflect the peculiar scenario of the neonatal brain, in which we showed random loss of transduced cells in the few days following LV transduction, coupled to moderate and physiological expansion of the subset of surviving vector-marked progenitor cells. Interestingly, the two most highly represented integration sites in this data set are in *alk* and *mtmr6* genes, which are expressed during CNS development and in postnatal brain tissues (56–59) and may thus be preferentially targeted by LV. Importantly, we never observed hyperproliferation of transduced cells or tumor mass formation, even long term after neonatal LV injection. This strongly suggests that the LV insertion does not provide selective advantage for survival and expansion in the neonatal brain. Interestingly, we found a unique clustering of integrations targeting *galc* exons only in bdLV.GALC-injected mice. Sequence analysis showed that the vector LTR and the *galc* exon sequences had the correct junction expected by an integration event. This result excluded the possibility that these integrations may result from homologous recombination between the nonintegrated bdLV.GALC sequence and *galc* exons. One possible explanation is that when multiple viral genomes enter the cell, the pre-integration complexes may integrate within each other and the host genome, creating false-positive integration sites. Further investigation will be required to clarify this unusual but interesting finding.

In summary, this study establishes the neonatal LV-mediated GT platform described here as a rapid, effective and safe therapeutic intervention that exploits endogenous neural cells as a long-lasting source of therapeutic enzyme. Also, it provides for the first time important information regarding the lack of detectable genotoxicity of this platform in the early postnatal CNS. This approach holds considerable significance to treat CNS pathology in GLD, MLD and possibly other devastating storage diseases and warrants validation in large animals before being considered for clinical evaluation.

## MATERIALS AND METHODS

### Animals

#### FVB/Twitcher mice

Twitcher mice (C57BL/6J background) bear a spontaneous point mutation resulting in a premature stop codon and no residual GALC activity (60). In this study, we used Twi mice on the mixed background of C57BL/6 and FVB generated by breeding C57BL/6 Twi (galc^+/−^) mice with WT (galc^+/+^) FVB mice (Jackson Laboratory, Bar Harbor, ME 04609 USA). FVB/Twi mice have been previously described (15,19). Tremors develop at around postnatal day (PND) 21 and progress to severe resting tremor, weight loss, paralysis, and wasting of hind legs. At 40 days, demyelination is observed in the PNS (severe) and CNS (moderate). Death occurs at ∼40 days. Homozygous (galc^−/−^) Twi mice and WT (galc^+/+^) littermates were obtained by brother–sister mating of heterozygous animals, and the genotyping of newborn mice was determined by polymerase chain reaction specific for the Twi mutation (61).

#### ARSA knockout mice

*As2^−/−^ or MLD mice* (62) cannot metabolize sulfatides. Starting at 6–7 months, they develop typical storage lesions, particularly in the fimbria and corpus callosum, and, to a lower extent in the PNS. Except for learning impairments, these mice do not show the severity of symptoms observed in MLD human patients and have a normal life expectancy.

#### NOD/SCID (NOD.CB17-Prkdc^scid^/NCrCrl)

The SCID mutation has been transferred onto a non-obese diabetic background. Animals homozygous for the SCID mutation have impaired T- and B-cell lymphocyte development (Charles River Laboratories, St Wilmington, MA 01887, USA).

Mouse colonies were maintained in the animal facility of the San Raffaele Scientific Institute, Milano, Italy. All procedures were performed according to protocols approved by the Internal Animal Care and Use Committee and were reported to the Ministry of Health, as per Italian law.

### Vector production

We used bidirectional lentiviral vectors (bdLV) allowing the dual-coordinate expression of two transgenes driven by the human phosphoglycerate kinase promoter (downstream cassette) and the minimal core promoter from cytomegalovirus (upstream cassette) (63). The ‘control’ vector (bdLV.CTRL) encodes for GFP and for the truncated form of the low-affinity nerve growth factor receptor (ΔNGFR). The ‘therapeutic’ vectors (bdLV.GALC and bdLV.ARSA) were generated by replacing the ΔNGFR cDNA of the CTRL vector with the murine GALC cDNA or ARSA cDNA C-terminally tagged with the human influenza hemagglutinin epitope (HA). VSV-pseudotyped third-generation LV were produced by transient four-plasmid co-transfection into 293T cells and purified by ultracentrifugation. Reagents, cloning procedures and sequence information are available upon request.

Expression titer of GFP was estimated on 293T cells by limiting dilution. Vector particle was measured by HIV-1 gag p24 antigen immunocapture (NEN Life Science Products, Zaventem, Belgium). Vector infectivity was calculated as the ratio between titer and particle for the vector expressing GFP or ΔNGFR. For all bdLVs, the expression titer of concentrate vector ranged between 1 and 3 × 10^9^ TU/ml and infectivity was in the range of 2 to 4 × 10^4^ TU/ng of p24.

### Intracerebral injection

#### Injection in the adult external capsule

WT mice from the FVB/Twi colony and As2^−/−^ mice (and their WT littermates) were injected with bdLV at PND21 and 6 months of age, respectively, and were euthanized 20 days or 6 months after injection. Surgery was performed as previously described (19). Viral suspension (2 × 10^6^ TU/1.5 µl) was slowly injected in the left hemisphere by means of a 33G needle Hamilton syringe (0.2 µl/min). Stereotactic coordinates (distance from Bregma in mm, according to the Paxinos mouse brain atlas): AP +1.4, ML +2.5, DV −3. Untreated As2^−/−^ mice and WT littermates were included as controls.

#### Injection in the neonatal external capsule

Newborn (PND2) Twi mice and WT littermates were anesthetized on crushed ice for 3 min and then placed on a refrigerated surgical table. A hole was made in the skull with a needle (without exposing the skull), and the viral suspension (2 × 10^6^ TU/1.5 µl) was slowly injected by means of glass capillary tightly connected to a 33G needle Hamilton mounted on a Micromanipulator M3310 (2Biological Instruments, Besozzo, VA, Italy). The capillary was left in place for additional 2 min and then slowly withdrawn. Stereotactic coordinates were as follows: anterior-posterior (AP) +1, medial-lateral (ML) +2, dorsal-ventral (DV) −1 (distance in mm from the frontal cerebral vein). Untreated Twi mice and WT littermates were included as controls. After the injection, mice were heated under a warming lamp, monitored until recovery and then returned to parental cures.

Survival after the neonatal and adult injection was >90%.

### Tissue collection and processing

Mice were euthanized at different time points after treatment (PND10, PND21, PND40, 6 and 12 months of age).

For biochemical and molecular assays, mice were euthanized with an overdose of avertine (500 mg/kg). The brain and the spinal cord (SC) were isolated and either quickly frozen in liquid nitrogen or immediately processed to obtain tissue extracts. Briefly, a cut was made to separate the Tel from the Cb, and a further incision was made to split the Tel in two hemispheres (injected and contralateral). Spinal cord was collected as a whole. The CSF was collected from the cysterna magna using a glass capillary. Blood was collected from the ophthalmic artery using a glass Pasteur pipette and left to clot for 30 min at room temperature. Serum was separated by centrifugation for 15 min at 12,000 g, collected and stored at −20°C.

For histopathology, immunohistochemistry and IF analysis, mice were deeply anesthetized with avertine (250 mg/kg) and intracardially perfused via the descending aorta with 0.9% NaCl followed by ice-cold 4% paraformaldehyde (PFA; P6148 Sigma–Aldrich, St. Louis, Mo, USA) in phosphate buffer solution (PBS). Brain and SC tissues were equilibrated for 24 h in 4% PFA, washed and stored in PBS + NaN_3_ at 4°C. Tissues were included in 4% agarose and serial coronal vibratome-cut sections (6 series, 40-μm-thick) were collected and stored in freezing solution (40% PBS, 30% ethylene glycol, 30% glycerol) before being processed as described below.

### Immunofluorescence

Free-floating vibratome-cut sections were incubated with blocking solution [10% normal goat serum (NGS) + 0.3% Triton X-100 in PBS] for 1 h at RT and then incubated overnight at 4°C with primary antibody diluted in blocking solution. After thorough washing (3 × 5 min each), antibody staining was revealed using species-specific fluorophore-conjugated secondary antibodies diluted in 10% NGS + 0.1% Triton X-100 in PBS.

For the detection of GALC, HA and Lamp1, tissue slices were incubated with 10% NGS + 0.5% Triton X-100 in PBS for 10 min before the incubation with the normal blocking solution. Incubation with primary antibodies was performed overnight at room temperature.

Coverslips and tissue sections were counterstained with dapi (4′,6-diamidino-2-phenylindole; 10236276001, Roche, Basel, Switzerland) or ToPro-3 (T3605, Life Technologies-Invitrogen, Carlsbad, CA, USA), washed in PBS and mounted on glass slides using Fluorsave (#345789, Calbiochem, Billerica, MA, USA).

### Immunohistochemistry

For the detection of Iba1, GFAP, CD68 and GFP, slices were fixed for 10 min in 3% H_2_O_2_ in PBS, washed in PBS, incubated for 30 min in blocking solution (0.3% Triton X-100 + 10% NGS in PBS) and then with primary antibodies. For the detection of GALC, brain slices were incubated with 10% NGS + 0.5% Triton X-100 in PBS for 10 min before the incubation with the normal blocking solution.

For the detection of lectins, slices were fixed for 10 min in 3% H_2_O_2_ in methanol, washed in PBS and incubated in Avidin solution (15 min) and Biotin solution (15 min)(Blocking kit; SP-2001, Vector Laboratories, Burlingame, CA 94010, USA). After washing, slices were incubated for 30 min in blocking solution (0.3% Triton + 10% NGS in PBS) and then with Biotinylated Ricinus Communis Agglutinin I (RCA I, B-1085 Vector Laboratories; 1 : 200) in blocking solution for 30 min.

Slices were then washed in PBS and incubated in Tris–HCl 100 mm (5 min), and staining was revealed using a biotinylated secondary antibody. The signal was enhanced incubating slices for 30 min with VECTASTAIN ABC kit (PK-6100, Vector Laboratories). After three washes in Tris–HCl 100 mm, the reaction with the substrate 3-3 diamino-benzidine tetrahydrochloride (DAB, 167 µg/ml in Tris–HCl 100 mm + H_2_O_2_ 1 : 3.000) was performed. Water was added to stop the reaction. Slices were washed in PBS and counterstained with cresyl violet (0.1% cresyl violet, 0.3% glacial acetic acid, H_2_O) for 2 min, then dehydrated and mounted with EUKITT (13155073, Bosch Sensortec GmbH, Reutlingen/Kusterdingen, Germany).

#### Primary antibodies

The primary antibodies used were mouse anti-APC (1 : 1,000; PO80 Calbiochem-EMD Millipore, Billerica, MA, USA), mouse anti-GFAP (1 : 1,000; MAB3402, Chemicon-Millipore, Temecula, CA, USA), rabbit anti-GFAP (1 : 1,000; ZO334, Dako, Denmark), mouse anti-HA (1 : 100; MMS-101P, Covance Princeton, NJ 08540, USA), mouse anti-NeuN (1 : 500; MAB377, Chemicon), rabbit anti-Iba1 (1 : 200; 019-19741, Wako Chemicals, Richmond, VA, USA), rabbit anti-Lamp-1 (1 : 200; AB 24170, Abcam Cambridge Science Park, Cambridge, UK), chicken anti-GFP (1 : 1,000; AB-13970, Abcam), rabbit anti-GFP (1 : 1000; A-11122 Molecular probes; Carlsbad, CA, USA), chicken anti-GALC (CL1021AP, 1 : 2,000; a gift of Dr C.W. Lee), rabbit anti-cleaved Caspase-3 (1 : 200, Cell Signalling, Danvers, MA, USA), rat anti-CD68 (1 : 100, MCA1957, AbD Serotec, Kidlington, Oxford, UK) and anti-CD3 (molecular complex; 1 : 80, BD Biosciences Pharmigen).

#### Secondary antibodies

Alexa 488-, Alexa 546-, Alexa 633- (Molecular Probes), Cy3- (Jackson ImmunoResearch Laboratories, West Grove, PA, USA) conjugated anti-rabbit, anti-mouse, anti-rat, anti-chicken antibodies (1 : 1,000); biotinilated goat anti-rabbit-, goat anti-rat-, goat anti-chicken-antibodies (1 : 200, Jackson ImmunoResearch Laboratories).

### Image acquisition

Slices processed for immunohistochemistry (GALC, lectins, GFAP, Iba1 and CD68) were visualized with a Nikon Eclipse E600 microscope. Images were acquired in bright field using a Nikon DMX 1200 digital camera and ACT-1 acquisition software (Nikon Corporation, Tokyo, Japan). Defined areas from the forebrain, Cb and SC were selected for the quantitative analysis, as previously described (19). Three to five pictures for each slice were taken, and the total immunopositive area in each picture (expressed in pixels) was calculated using the ImageJ software. Tissue slices from UT WT mice (for lectins and CD68) or slices in which the primary antibodies were omitted (for GFAP and Iba1) were used to set the signal threshold.

Slice processed for IF were visualized with: (1) Zeiss Axioskop2 microscope using double laser confocal microscopy with Zeiss Plan-Neofluar objective lens (Zeiss, Arese, Italy). Images were acquired using a Radiance 2100 camera (Bio-Rad, Segrate, Italy) and LaserSharp 2000 acquisition software (Bio-Rad); (2) Perkin Elmer UltraVIEW ERS Spinning Disk Confocal (PerkinElmer Life Sciences, Inc., MA, USA), 63×/NA1.4 Planapochromat oil-immersion lens (Carl Zeiss, Jena, Germany) with a 405-nm diode laser, 488-nm argon laser and a 568-nm krypton laser excitation wavelengths.

Co-localization between GFP and microglial markers (Iba1, CD68-positive cells) was analyzed on sequential confocal images (*Z-*stacks) collected at 0.25-μm intervals covering 3-μm depth (12 total scanning images for each channel). The 3D signals and the orthogonal projections representation were obtained by means of Volocity Software (v5.2.1; PerkinElmer-Improvision, Lexington, MA, USA).

3D Reconstruction and quantification of the bdLV-transduced brain tissue were performed using a Leica DM 4000B microscope attached to a Neurolucida computer-assisted tracing system (Microbrightfiled, Inc., USA). For calculation of GFP-transduced volume, every GFP-positive area on serial coronal sections (40-μm-thick, 240-μm-interval; *n* = 12 sections from one series, collected from Bregma −2 mm to +1 mm) was marked and attached to the subsequent trace according to the software instructions.

Images were imported into Adobe Photoshop CS4 (USA) and adjusted for brightness and contrast.

### Cell counts

Quantification was performed in matched coronal brain sections (3–6 slices/mice; *n* = 3–4 mice/group) selected within the region containing transduced (GFP^+^) cells (identified using anti-GFP antibody) in combination with lineage-specific markers (NeuN, neurons; GFAP, astroglia; APC, oligodendroglia; Iba1, microglia; CD68, macrophages), apoptosis (Casp3) and cell proliferation (Ki67) markers and nuclear counterstaining. Triple-labeled sections were scanned with the confocal laser microscope at 40× magnification and a representative sample of 100–200 transduced (GFP^+^) cells showing defined nuclei was examined for co-localization with either marker. Results were expressed as follows: (1) percentage of GFP^+^ cells on total nuclei (efficiency of transduction), (2) percentage of GFP^+^marker^+^ cells within the GFP^+^ cells and (3) percentage of marker^+^ cells on total nuclei.

### Detection of LV genome

Genomic DNA was extracted from murine brain tissues (#200600, DNA Extraction Kit, Agilent Technologies, Inc. Santa Clara, CA 95051, USA) and from liver, spleen and gonads (#13343 Blood and Cell Culture DNA Midi Kit, Qiagen, Venlo, 5911 KJ, Netherlands). Samples were quantified at NanoDrop ND-1000 Spectrophotometer (Euroclone, Pero, Italy). Vector copies per genome were quantified by TaqMan analysis as previously described (19). Briefly, quantitative PCR was performed starting from 100 ng of template DNA by amplifying the PSI (ψ) sequence of the LV backbone and a fragment of the murine β-actin gene. A standard curve of genomic DNA carrying 4 LV copies, validated by Southern blot analysis, was constructed using DNA extracted from transgenic mouse tissue. The standard curve, based on different dilutions of DNA (from 200 to 25 ng), and accordingly, of LV copies, was used as standard both for LV and for β-actin amplification. Reactions were carried out in a total volume of 25 µl, in an ABI Prism 9700 HT Sequence Detection System (Life Technologies-Applied Biosystems, Carlsbad, CA, USA). The VCN was calculated as follows: (ng LV/ng endogenous DNA) × (number of LV integrations in the standard curve). Primers and probes are available on demand.

### Detection of GALC mRNA by RT–PCR

Total RNA was isolated from cells and tissues using Trizol Reagent (Invitrogen)/Chloroform method and purified by RNeasy mini Kit (Qiagen, Hilden, Germany). The quantity of RNA was determined by 260/280 nm optical density reading on a NanoDrop ND-1000 Spectrophotometer (NanoDrop, Pero, Italy). Reverse transcription was carried out using 1 μg of total RNA in the presence of 200 U of Quantitect Reverse Transcriptase kit (Qiagen, Hilden, Germany). PCR was carried out using Taq DNA Polymerase (Qiagen) according to manufacturer's protocol. Galc endogenous mRNA was amplified using forward and reverse primers into the mouse gene sequence (NM_008079), whereas for the transgenic one, we designed a reverse primer into the vector construct WPRE sequence (forward: 5′ GAC TGT GCA GTG CGA TGT TT 3′; reverse Galc: 5′ GCC CTG AAC CAA AAT CAA AA 3′, reverse WPRE: 5′ AAG CCA TAC GGG AAG CAA TA 3′; annealing temperature, 56°C). Expected product size is 437 bp (endogenous) and 550 bp (transgenic).

All cDNAs used as templates were previously normalized throughout β-actin RT–PCR. The PCR products were separated on a 2% agarose gel at 10 V/cm and visualized by ethidium bromide.

### Determination of enzymatic activity

GALC activity was measured using the artificial fluorogenic substrate 4-methylumbelliferone-β-galactopyranoside. ARSA activity was determined using the 4-methylumbelliferyl-sulfate substrate and calculated by subtracting the value obtained in the presence of AgNO_3_ [Arylsulphatase B (ARSB) activity] from that measured in the absence of the inhibitor (ARSA + ARSB activity). Both assays were performed according to previously described protocols (64,65) that have been validated in our previous studies (15,19).

### Psychosine quantification

Tissues were stored at −80°C. For homogenization (using a TissueLyser), samples were combined with 5–10 times their volume of PBS and 20 µl of the resulting homogenate was used for determination of protein content (BCA method). ^2^H_7_-psychosine (AVANTI Polar Lipids, Inc.) was added to brain homogenates (1 mg of protein) as internal standard. After deproteinization and extraction by methanol/chloroform (1 : 1) and evaporation to dryness (N_2_, 60°C) of the organic layer, the sample extract was reconstituted in methanol. After injection and elution over a C8 analytical column, psychosine and its internal standard were detected by positive ESI in the MRM mode on a TSQ Quantum AM mass spectrometer (Thermo). Psychosine concentrations were established by the use of calibration standards. Concentrations were expressed per 1 mg of protein.

### Detection of anti-GFP and anti-HA antibodies

Sera collected from UT and bdLV-treated mice were tested for the presence of anti-GFP and anti-HA antibodies via enzyme-linked immunosorbent assay (ELISA). Microtiter plates were coated with recombinant-GFP (Vector Laboratories) or HA tag peptide (Abcam), 0.3 μg/well in 1 m carbonate buffer, pH 9.5. After three washes in PBS + 0.05% Tween-20, wells were incubated with blocking buffer (PBS + 0.05% Tween-20 + 1% goat serum) for 2 h at RT in the dark. Serial dilutions in PBS + 0.05% Tween-20 (from 1 : 10^2^ to 1 : 10^5^) of sera were added for 2 h at RT in the dark. Following four washes, anti-GFP and anti-HA antibodies were detected by adding 100 µl/well of diluted (1 : 2.000) peroxidase-conjugated rabbit anti-mouse Ig (Dako Cytomation) for 1 h at RT in the dark. After four washes, plates were reacted with OPD (Sigma–Aldrich) and H_2_O_2_ (final concentration 0.03%). The reaction was stopped by adding 50 µl/well of 1 m sulfuric acid. Plates were read using an ELISA reader (VERSAmax Molecular Devices, Sunnyvale, CA, USA), and values are expressed as optical density (OD; λ 492 nm).

Twi mice immunized by systemic injection of 3 × 10^8^ TU/150 µl of bdLV.GALC were used as positive controls (injection at PND21 and collection of sera at PND42; OD = 2.149 with 1 : 10^3^ dilution). Anti-HA monoclonal antibody was used as positive control for anti-HA antibodies detection (OD = 2.643 with 1 : 10^3^ dilution). Sera of UT or PBS-injected mice were used as negative controls. Threshold was set as the average OD measured in UT mice plus three standard deviations (OD = 0.307 and 0.273 for GFP and HA, respectively).

### P24 enzyme-linked immunosorbent assay

The presence of vector particle in the sera of treated mice and controls was determined by immunosorbent assay, using the Alliance HIV-1 p24 antigen ELISA kit (PerkinElmer), following manufacturer's instructions. Serial dilutions of sera were plated in triplicate in microplate wells, coated with a mouse monoclonal antibody to the HIV-1 capsid protein p24, and vector particle in the sera was lysed by 5% Triton X-100. The captured antigen was then complexed with biotinilated polyclonal antibody to HIV-1 p24, followed by a streptavidin–HRP (horseradish peroxidase) conjugate. The resulting complex was detected by incubation with ortho-phenylenediamine-HCl, which produces a yellow color that is directly proportional to the amount of HIV-1 p24 captured. The absorbance of each microplate well was determined using a microplate reader (Versa Max, Molecular Devices) (λ 490 nm), and p24 amount was calculated using an HIV-1 p24 antigen standard curve.

### LAM-PCR and 454-pyrosequencing

The procedures and primer sequences for LAM-PCR for LV integration site retrieval have been previously described (66). Briefly, we used 1 μg of DNA extracted from brain tissues as template for 100-cycle linear PCR preamplification of genome–vector junctions, followed by magnetic capture of the biotinylated target DNA, hexanucleotide priming, restriction digest using Tsp509I and HpyCHIV4 and ligation of a restriction site–complementary linker cassette. The first exponential biotinylated PCR product was captured via magnetic beads and reamplified by a nested second exponential PCR. We separated LAM-PCR amplicons on high-resolution Spreadex gels (Elchrom Scientific) to evaluate PCR efficiency and the bands pattern for each sample. We adapted the LAM-PCR samples for 454-pyrosequencing by fusion-primer PCR to add the Roche 454 GS-FLX adaptors (41): adaptor A, plus a 6-nucleotide barcode was added at the LTR end of the LAM-PCR amplicon; adaptor B was added at the linker cassette side. Each sample was amplified with fusion primers carrying each a different barcode sequence. Fusion-primer PCR products were assembled in equimolar ratio into libraries, avoiding repetition of identical barcodes, and sequenced at GATC biotech. Sequences were aligned to the mouse genome (assembly July 2007, 9 mm) using the UCSC genome browser (http://genome.ucsc.edu/), coupled to bioinformatic analyses. Genes targeted by vector integrations were considered those nearest to the integration site.

### Statistics

Data were analyzed with Graph Pad Prism version 5.0a for Macintosh and expressed as mean ± SEM (with *n* ≥ 3) if not otherwise stated. Non-parametric (Kruskall–Wallis) or parametric tests (Student's *t*-test, one-way or two-way analysis of variance) followed by post-tests were used according to data sets. Survival curves were analyzed by Log-Rank (Mantel–Cox) test. A *P*-value of <0.05 was considered statistically significant. The number of samples analyzed and the statistical test used are indicated in the legends to each figure.

## SUPPLEMENTARY MATERIAL

Supplementary Material is available at *HMG* online.

## FUNDING

This work was funded by Comitato Telethon Fondazione Onlus; grants numbers TGT06B02 and TGT11B02 to A.G. and the European Union's Seventh Framework Programme (FP7/2007-2013) under grant agreement number 241622. Funding to pay the Open Access publication charges for this article was provided by Comitato Telethon Fondazione Onlus.

## Supplementary Material

Supplementary Data
