# Supplementary material for: Therapeutic benefit of lentiviral-mediated neonatal intracerebral gene therapy in a mouse model of globoid cell leukodystrophy
Source: Hum Mol Genet. 2014 Jan 23;23(12):3250–68. doi: 10.1093/hmg/ddu034 (PMC4030779; doi:10.1093/hmg/ddu034)
Supplement: Supplementary Data [file supp_23_12_3250__index.html]

Therapeutic benefit of lentiviral-mediated neonatal intracerebral gene therapy in a mouse model of globoid cell leukodystrophy — Therapeutic benefit of lentiviral-mediated neonatal intracerebral gene therapy in a mouse model of globoid cell leukodystrophy — Supplementary Data 

# Therapeutic benefit of lentiviral-mediated neonatal intracerebral gene therapy in a mouse model of globoid cell leukodystrophy

## Supplementary Data

Supplementary Data

**Files in this Data Supplement:**

- Supplementary Data - Pdf file
- Supplementary Table 1 - pdf file
- Supplementary Table 2 - pdf file
