# Supplementary material for: Therapeutic benefit of lentiviral-mediated neonatal intracerebral gene therapy in a mouse model of globoid cell leukodystrophy
Source: Hum Mol Genet. 2014 Jan 23;23(12):3250–68. doi: 10.1093/hmg/ddu034 (PMC4030779; doi:10.1093/hmg/ddu034)

Figure S1. High resolution Spreadex gel electrophoresis of LAM-PCR products done on brain tissues from mice injected at PND2 and PND21. We used the restriction enzymes Tsp509I (A) and HpyCH4 (B). DNA extracted from an untrasduced tissue was used as negative control (C-). DNA extracted from a transduced CEM cell clone was used as positive control (C+). M, marker. Asterisks indicate spillover. VCN values for each sample are indicated at the bottom of each lane (see also Table 1).

Lattanzi et al., Fig. S1

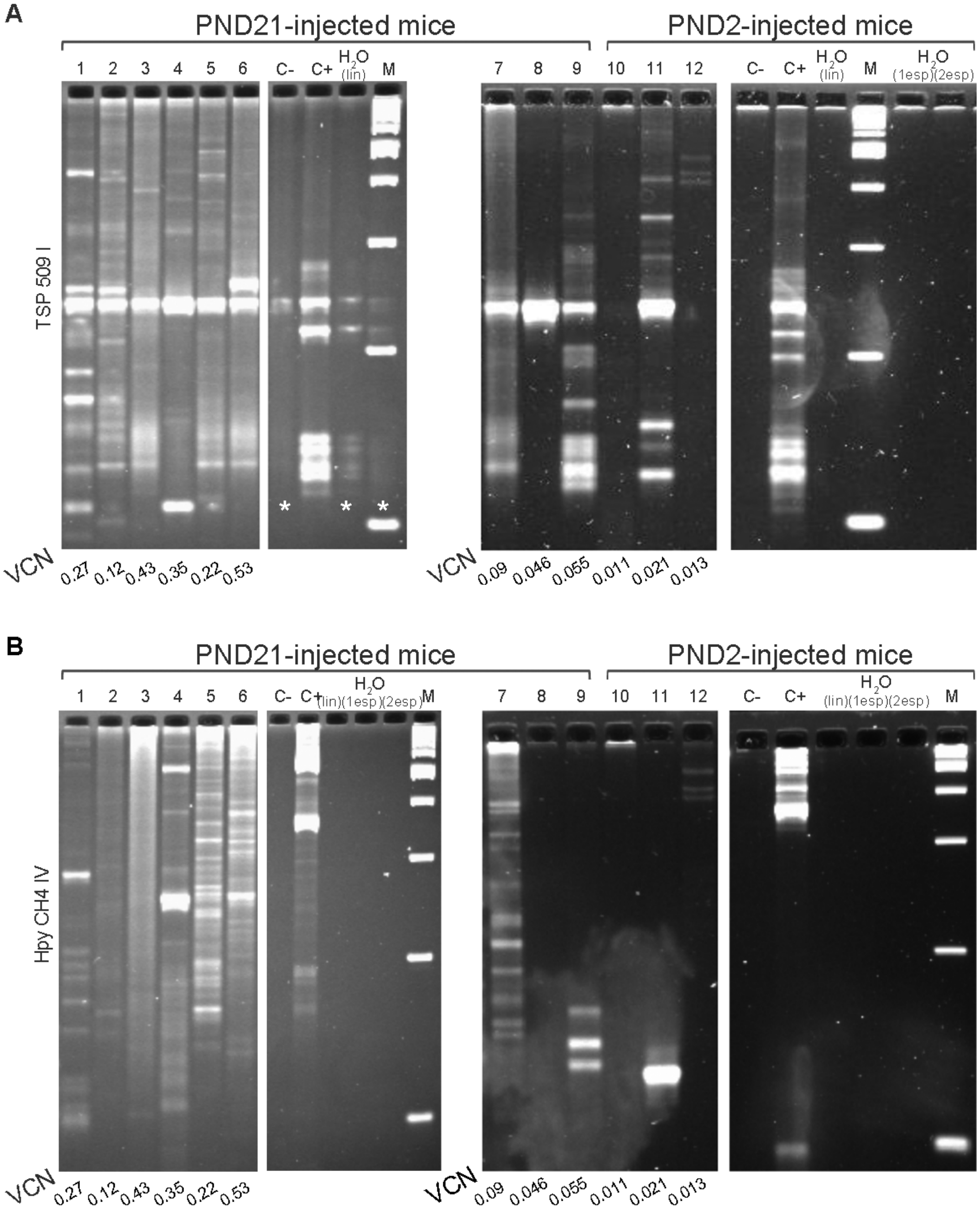

Supplement: Supplementary Data [file supp_ddu034_ddu034supp.pdf]
