# Supplementary material for: Therapeutic benefit of lentiviral-mediated neonatal intracerebral gene therapy in a mouse model of globoid cell leukodystrophy
Source: Hum Mol Genet. 2014 Jan 23;23(12):3250–68. doi: 10.1093/hmg/ddu034 (PMC4030779; doi:10.1093/hmg/ddu034)
Supplement: Supplementary Data [file supp_ddu034_ddu034supp_table1.pdf]

Table S1. Clonal abundance calculated as the proportion of sequencing reads (% of reads) representing each integration site within each datasets (PND2 injection and PND21 injection). The number of sequence counts for each targeted gene is indicated. All vector integrations with an abundance of <1% have been grouped.

**PND21 injection**			**PND2 injection**		
% of reads	gene	Sequence count	% of reads	gene	Sequence count
8.550365581	Mamdc2	1415	42.73011295	Mtmr6	1778
5.08792072	F2rl3	842	13.19394376	Alk	549
4.296332105	Basp1	711	4.15765441	Ccdc130	173
3.299293009	Bdh2	546	2.667627974	Rps6ka2	111
2.912562693	Tmem65	482	2.355203076	Pam	98
2.163272705	Pkia	358	2.211006969	Nr2f1	92
2.060547465	Mdga2	341	2.114876232	Fzd1	88
1.643603843	Smarca1	272	2.042778178	Lrrc9	85
1.595262554	Nudt3	264	1.970680125	Lrrc7	82
1.480451991	Fam76b	245	1.153568854	Mtor	48
1.462324007	Megf9	242	1.033405431	Pcgf3	43
1.450238685	Tbc1d19	240	1.033405431	Inpp4b	43
1.42606804	Nt5c3	236	*23.3357366*	*all <1%*	*971*
1.359598767	Diap3	225			
1.341470784	Hibadh	222			
1.299172155	Slitrk1	215			
1.232702882	Fam53b	204			
1.111849659	Zfp667	184			
1.087679014	Cd200r4	180			
1.075593691	Plscr2	178			
1.003081757	Asph	166			
53.06060789	*all <1%*	*8781*			
Total reads		16549	Total reads		4161
