# Supplementary material for: Therapeutic benefit of lentiviral-mediated neonatal intracerebral gene therapy in a mouse model of globoid cell leukodystrophy
Source: Hum Mol Genet. 2014 Jan 23;23(12):3250–68. doi: 10.1093/hmg/ddu034 (PMC4030779; doi:10.1093/hmg/ddu034)
Supplement: Supplementary Data [file supp_ddu034_ddu034supp_table2.pdf]

Table S2. Gene Ontology (GO) analysis of genes targeted by LV integrations following intracerebral injection.

The datasets of genes targeted by LV integrations in brain tissues from mice injected with LVs at postnatal day (PND)2 (**A**; 172 genes) or PND21 (**B**; 822 genes) were analyzed by the online software DAVID EASE (http://david.abcc.ncifcrf.gov) to score for the significant enrichment of specific gene classes. The GO terms chosen for this analysis were “Biological process” and “Molecular Function”. Count: number of genes in the dataset that belong to the given gene class; P value: only P<0.05 were considered; Fold Change: only fold changes >2 were considered; Benjamini: P value after Benjamini correction for repeated measures. Given the relatively large number of genes analyzed in B, only gene classes with P <0.05 after Benjamini correction are shown.

A) PND2 injection

GO term	Gene Class	Count	P Value	Fold Change	Benjamini
Biological process	nervous system development	19	5.7E-04	2.5	4.2E-01
	regulation of small GTPase mediated signal transduction	9	1.0E-03	4.4	3.9E-01
	regulation of cell communication	16	3.6E-03	2.3	4.4E-01
	regulation of Ras GTPase activity	5	5.7E-03	6.9	5.0E-01
	regulation of Ras protein signal transduction	7	5.7E-03	4.3	5.4E-01
	regulation of signal transduction	14	6.2E-03	2.4	4.5E-01
	synaptogenesis	3	8.8E-03	21	5.1E-01
	negative regulation of nucleobase, nucleoside, nucleotide and nucleic acid metabolic process	10	9.2E-03	2.8	5.0E-01
	synapse organization	4	9.5E-03	9.1	4.8E-01
	regulation of GTPase activity	5	9.6E-03	6	4.6E-01
	negative regulation of nitrogen compound metabolic process	10	9.8E-03	2.8	4.5E-01
	negative regulation of cellular metabolic process	11	1.2E-02	2.5	5.0E-01
	germ cell development	5	1.3E-02	5.5	4.8E-01
	negative regulation of macromolecule biosynthetic process	10	1.3E-02	2.7	4.9E-01
	negative regulation of cellular biosynthetic process	10	1.5E-02	2.6	5.2E-01
	taxis	5	1.6E-02	5.1	5.2E-01
	chemotaxis	5	1.6E-02	5.1	5.2E-01
	negative regulation of biosynthetic process	10	1.6E-02	2.6	5.2E-01
	negative regulation of transcription	9	1.8E-02	2.7	5.4E-01
	cell-cell adhesion	7	1.9E-02	3.3	5.4E-01
	negative regulation of metabolic process	11	2.1E-02	2.3	5.5E-01
	positive regulation of cell-substrate adhesion	3	2.4E-02	12	5.8E-01
	negative regulation of gene expression	9	3.1E-02	2.4	6.4E-01
	cellular component morphogenesis	8	3.8E-02	2.5	6.9E-01
	negative regulation of macromolecule metabolic process	10	3.8E-02	2.2	7.0E-01
	neurotransmitter secretion	3	4.1E-02	9.3	7.0E-01
	extracellular structure organization	5	4.5E-02	3.7	7.2E-01
	locomotion	8	4.5E-02	2.4	7.2E-01
	oogenesis	3	4.7E-02	8.5	7.3E-01
Molecular function	GTPase activator activity	7	4.8E-03	4.5	3.1E-01
	GTPase regulator activity	9	9.2E-03	3	3.8E-01
	small GTPase regulator activity	7	9.7E-03	3.8	3.5E-01
	nucleoside-triphosphatase regulator activity	9	1.0E-02	3	3.2E-01
	enzyme activator activity	7	1.6E-02	3.4	3.9E-01
	small GTPase regulator activity	7	9.7E-03	3.8	3.5E-01
	hydrolase activity, acting on ester bonds	12	2.3E-02	2.1	4.2E-01
	transcription cofactor activity	6	1.6E-02	4.1	4.1E-01
	transcription repressor activity	6	2.9E-02	3.5	4.7E-01
	transcription factor binding	7	3.0E-02	3	4.6E-01

B) PND21 injection

GO term	Gene Class	Count	P Value	Fold Change	Benjamini
Biological process	cell adhesion	53	2.8E-08	2.3	1.6E-05
	biological adhesion	53	3.0E-08	2.3	1.4E-05
	cell-cell adhesion	25	3.8E-05	2.6	3.3E-03
	nervous system development	78	3.4E-11	2.2	7.9E-08
	generation of neurons	49	2.8E-08	2.4	2.1E-05
	neuron differentiation	42	5.5E-08	2.6	2.1E-05
	neurogenesis	50	1.2E-07	2.3	3.9E-05
	neuron development	34	1.2E-07	2.8	3.6E-05
	cell morphogenesis involved in neuron differentiation	24	1.5E-06	3.2	2.7E-04
	cell projection morphogenesis	25	2.7E-06	3.0	4.5E-04
	neuron projection morphogenesis	23	3.1E-06	3.2	4.7E-04
	axonogenesis	22	3.1E-06	3.3	4.5E-04
	neuron projection development	26	3.2E-06	2.9	4.4E-04
	cell morphogenesis	32	3.9E-06	2.5	4.7E-04
	cell part morphogenesis	25	6.4E-06	2.9	6.9E-04
	cell morphogenesis involved in differentiation	25	6.4E-06	2.9	6.9E-04
	axon guidance	16	1.0E-05	4.0	1.0E-03
	cellular component morphogenesis	33	2.0E-05	2.3	2.0E-03
	cell projection organization	30	5.1E-05	2.3	4.0E-03
	cell motion	31	2.5E-04	2.1	1.9E-02
	nervous system development	78	3.4E-11	2.2	7.9E-08
	generation of neurons	49	2.8E-08	2.4	2.1E-05
	neuron differentiation	42	5.5E-08	2.6	2.1E-05
	neurogenesis	50	1.2E-07	2.3	3.9E-05
	cell motion	31	2.5E-04	2.1	1.9E-02
Molecular function	potassium channel activity	16	3.1E-04	3.0	2.9E-02
